# A Concise Total Synthesis of the Fungal Isoquinoline Alkaloid TMC-120B

**DOI:** 10.3390/molecules27020521

**Published:** 2022-01-14

**Authors:** Ahmad K. Haidar, Niels D. Kjeldsen, Nikolaj S. Troelsen, Viola Previtali, Kasper P. Lundquist, Thomas O. Larsen, Mads H. Clausen

**Affiliations:** 1 Center for Nanomedicine and Theranostics, Department of Chemistry, Technical University of Denmark, Kemitorvet 207, 2800 Kongens Lyngby, Denmark; ahmhai@biosustain.dtu.dk (A.K.H.); nikolaj.s.troelsen@outlook.com (N.S.T.); Viola.Previtali@iit.it (V.P.); kaslun@kemi.dtu.dk (K.P.L.); 2 NCK A/S, Rugmarken 28, 3520 Farum, Denmark; ndk@nck.dk; 3 Department of Biotechnology and Biomedicine, Technical University of Denmark, Søltofts Plads, Building 221, 2800 Kongens Lyngby, Denmark; tol@bio.dtu.dk

**Keywords:** isoquinolines, alkaloids, epilepsy

## Abstract

Recent reports of antiepileptic activity of the fungal alkaloid TMC-120B have renewed the interest in this natural product. Previous total syntheses of TMC-120B comprise many steps and have low overall yields (11–17 steps, 1.5–2.9% yield). Thus, to access this compound more efficiently, we herein present a concise and significantly improved total synthesis of the natural product. Our short synthesis relies on two key cyclization steps to assemble the central scaffold: isoquinoline formation via an ethynyl-imino cyclization and an intramolecular Friedel-Crafts reaction to form the furanone.

## 1. Introduction

**Figure 1:**
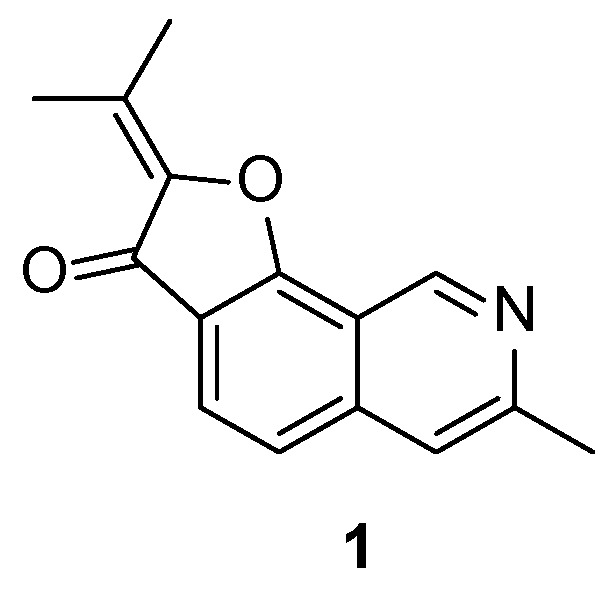
The fungal natural product TMC-120B (**1**).

The fuoroisoquinoline alkaloid TMC-120B (**1**) was first isolated from the fungus *Aspergillus ustus* by Kamutsubara et al. in 1999 (Figure 1) [1]. TMC-120B was initially discovered as a modulator of interleukin-5 mediated eosinophil viability [2], but has recently been reported to also exhibit antiepileptic activity in a Zebrafish model [3]. Epilepsy describes a group of serious neurological disorders that are characterized by recurrent epileptic seizures [4,5] and has been estimated to affect as many as 50 million people worldwide in 2016 [6]. While many patients experience lasting seizure remissions with present antiepileptic drugs (e.g. sodium valproate, lamotrigine, topiramate, and carbamazepine), [5,7] about one in three patients remain insensitive to current treatment options (drug-resistant epilepsy) [7,8]. Furthermore, TMC-120B is non-cytotoxic in human cancer cell lines [9] and has shown activity in the 6 Hz psychomotor mouse model for epilepsy, [10] suggesting that further biological investigations are warranted [11].

**Scheme 1:**
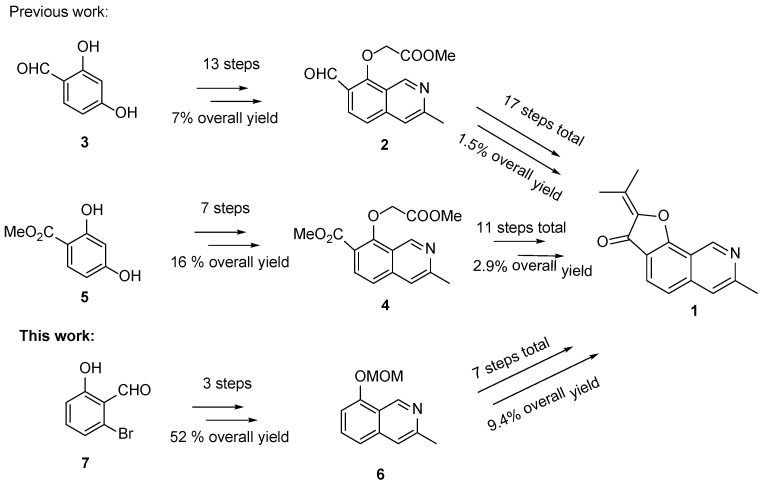
Two previously reported total syntheses of TMC-120B (**1**) [12,14] and this work.

With renewed interest in the biological properties of **1**, a need for convenient access to this natural product has emerged [3]. The first total synthesis of **1** was reported by Hibino et al. in 2003 and consisted of 17 steps from 2,4-dihydroxybenzaldehyde (**3**) with an overall yield of 1.5% [12,13]. In 2012, the same group reported an improved total synthesis of **1** in 11 steps starting from methyl β-resorcylate (**5**) with an overall yield of 2.9% (Scheme 1) [14]. However, in order to access larger quantities of this natural product more efficiently, and potentially analogues thereof, a shorter and more efficient route is required. Thus, we herein present a concise and significantly improved total synthesis of **1** (Scheme 1). Moreover, in the first total synthesis, the isoquinoline framework **2** was constructed from **3** in 11 steps (7% overall yield), improved by the same group by constructing **4** from **5** in seven steps (16% overall yield); in our work, we simplified the synthesis of isoquinoline framework **6** into three steps (52% overall yield).

## 2. Results and Discussion

**Scheme 2:**
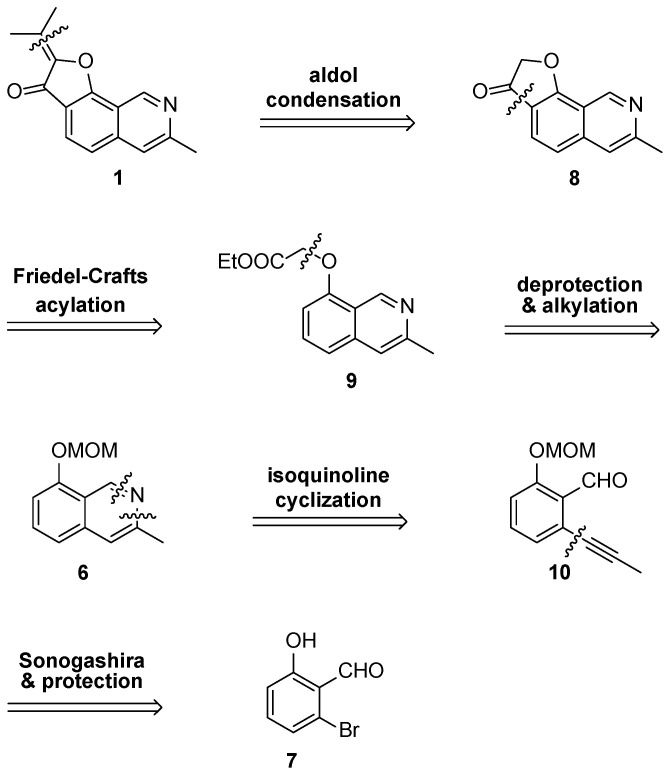
Retrosynthetic analysis of **1** showing two key cyclization steps—intramolecular Friedel-Crafts acylation and isoquinoline formation.

Total synthesis of **1** was envisioned using two key cyclization steps for assembly of the central scaffold. An intramolecular Friedel-Crafts reaction was planned to form the final furanone ring while an ethynyl–imine cyclization was selected to form the isoquinoline scaffold (Scheme 2).

**Scheme 3:**
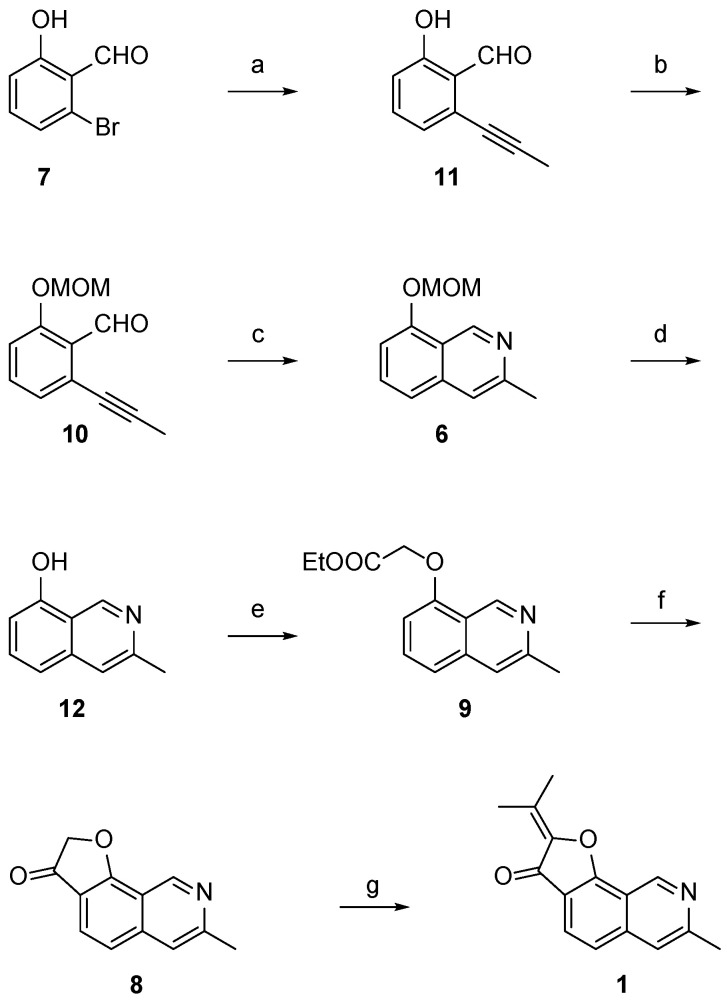
Total synthesis of TMC-120B (**1**). Reagents and conditions: (**a**) propyne, PdCl_2_(PPh_3_)_2_, CuI, Et_3_N, 55 °C, 24 h, 69%; (**b**) MOMCl, DIPEA, CH_2_Cl_2_, 40 °C, 16 h, 84%; (**c**) NH_3_, 80 °C, 7 h, 89%; (**d**) HCl, MeOH, reflux, 3 h, 90%; (**e**) ethyl bromoacetate, NaOEt, DMF, 12 h, 73%; (**f**) *i.* NaOH, EtOH, 1 h, *ii*. SOCl_2_, reflux, 1 h, *iii*. AlCl_3_, 1 h, 79%; (g) *p*-TsOH·H_2_O, acetone, DCE, reflux, 5 h, 35%.

The synthetic route to access **1** is shown in Scheme 3. From readily available 2-bromo-6-hydroxybenzaldehyde (**7**), an initial Sonogashira coupling [15,16] with propyne was accomplished to afford **11** at a 69% yield. Initial attempts to form the isoquinoline scaffold from **10** were unsuccessful both via conversion of **10** into the corresponding *tert*-butylimine followed by a copper-catalyzed imino-annulation [17] or attempting a tandem imine formation and subsequent ethynyl–imino cyclization reaction with ammonia [18]. Methoxymethyl acetal (MOM) protection of the phenol was then performed to generate intermediate **8** in 84% yield [19]. While imino-annulation via the *tert*-butylimine still did not occur, formation of the isoquinoline scaffold was accomplished using the tandem imine and ethynyl–imino cyclization sequence to afford **6** at an 89% yield. This presents a significant improvement over previous efforts to synthesize the core isoquinoline scaffold of **1** via thermal aza-electrocyclic reactions (46% yield) [14]. The isoquinoline cyclization was also carried out by formation of the corresponding *O*-allyl oxime of **10** and a subsequent silver trifluoro-methane-sulfonate/triflic acid (AgOTf/TFOH) co-catalyzed redox reaction, but only provided **6** at a 48% yield over two steps [20].

MOM deprotection under acidic conditions proceeded at a 90% yield to afford isoquinolinol **12**. This enabled alkylation of the phenol with ethyl bromoacetate to give the second key intermediate **9** in 73% yield. Instead of a Dieckmann condensation [21] and decarboxylation sequence to form the final furanone ring as previously reported, [12,13,14] cyclization was realized using an intramolecular Friedel-Crafts acylation [22]. Hydrolysis to the carboxylic acid and subsequent conversion to the corresponding acid chloride was achieved with sodium hydroxide (NaOH) and thionyl chloride (SOCl_2_), followed by the addition of aluminum trichloride to assemble the complete scaffold of TMC-120B (**1**) in 79% yield over two steps. The total synthesis of **1** was then finalized using an aldol condensation between **8** and acetone under acidic conditions at a 35% yield. Comparison of NMR spectroscopic data with that of previous reports and isolation from natural extracts confirmed the identity of **1** [1,13].

## 3. Experimental

### 3.1. General Methods

Commercially available reagents were used without further purification and all solvents were of HPLC quality. Unless otherwise stated, reactions were carried out under an atmosphere of nitrogen and heating was performed in an oil bath. Reactions were monitored by thin layer chromatography (TLC) and/or reversed-phase ultra-performance liquid chromatography mass spectrometry (RP-UPLC-MS). Analytical TLC was conducted on Merck aluminum sheets covered with silica (C60). The plates were either visualized under UV-light or stained by dipping in a developing agent followed by heating. Potassium permanganate KMnO_4_ (3 g in water (300 mL) along with potassium carbonate K_2_CO_3_ (20 g)) was used as developing agent. Flash column chromatography was performed using Merck Geduran^®^ Si 60 (40–63 µm) silica gel. Analytical RP-UPLC-MS (ESI) analysis was performed on a S2 Waters AQUITY RP-UPLC system equipped with a diode array detector using an Thermo Accucore C18 column (d 2.6 µm, 2.1 × 50 mm; column temp: 50 °C; flow: 1.0 mL/min). Eluents A (10 mM NH_4_OAc in H_2_O) and B (10 mM ammonium acetate NH_4_OAc in acetonitrile MeCN) were used in a linear gradient (5% B to 100% B) for 2.4 min and then held for 0.1 min at 100% B (total run time: 2.6 min). The LC system was coupled to a SQD mass spectrometer. All new compounds were characterized by ^1^H NMR, ^13^C NMR, IR, HRMS (ESI), and melting point.

NMR data were acquired at 298 K using a 400 MHz Bruker AVANCE III HD spectrometer equipped with a Prodigy CryoProbe. The chemical shifts (δ) are reported in parts per million (ppm) and the coupling constants (*J*) in Hz. For spectra recorded in dimethyl sulfoxide DMSO-*d*_6_, chemical shifts are reported relative to the signal for DMSO-*d*_5_ (δ 2.50 ppm for ^1^H NMR and δ 39.52 ppm for ^13^C NMR). For spectra recorded in CDCl_3_, chemical shifts are reported relative to the signal for CHCl_3_ (δ 7.26 ppm for ^1^H NMR and δ 77.16 ppm for ^13^C NMR). For spectra recorded in D_2_O, ^1^H chemical shifts are reported relative to the signal for HDO (δ 4.79 ppm for ^1^H NMR) and ^13^C chemical shifts are reported relative to the signal for DMSO-*d*_6_ (added as an internal standard). NMR data was analyzed using MestReNova (v11.0.0-17609) by Mestrelab Research S.L. All NMR Spectra for compounds **1**, **5**, **6** hydrolyzed, **7**, **8**, **9** and **10** can be found in Supplementary Materials.

IR analysis was performed on a Bruker Alpha FT-IR spectrometer. Melting points were obtained using a Stuart SMP30 melting point apparatus and are uncorrected. Analytical LC-HRMS (ESI) analysis was performed on an Agilent 1100 RP-LC system or a Waters Alliance 2695. The Agilent 1100 was equipped with a diode array detector using a Phenomenex Luna C18 column (d 3 µm, 2.1 × 50 mm; column temp: 40 °C; flow: 0.4 mL/min). Eluents A (0.1% HCO_2_H in H_2_O) and B (0.1% formic acid HCO_2_H in MeCN) were used in a linear gradient (20% B to 100% B) in a total run time of 15 min. The LC system was coupled to a Micromass LCT orthogonal time-of-flight mass spectrometer equipped with a Lock Mass probe operating in positive electrospray mode. The Waters Alliance 2695 was equipped with a diode array detector without a column. Eluents A (0.1% HCO_2_H in H_2_O) and B (0.1% HCO_2_H in MeCN) were used as a 1:1 mixture in a linear gradient with a total run time of 3 min. The LC system was coupled to a Micromass LCT Premier XE operating in positive electrospray mode. All IR spectra for compounds **1**, **5**, **6** hydrolyzed, **7**, **8**, **9** and **10** can be found in Supplementary Materials.

### 3.2. 2-Hydroxy-6-(prop-1-yn-1-yl)benzaldehyde *(**11**)*

In a 3-necked, N_2_-flushed, 6 L reactor equipped with mechanical stirrer, condenser and heating mantle was mixed triethylamine (NEt_3_) (320 mL, degassed with N_2_) and 2-bromo-6-hydroxybenzaldehyde (20.5 g, 102 mmol). The mixture was heated to 41 °C and Bis(triphenylphosphine)palladium chloride (Pd(PPh_3_)_2_Cl_2_) (3.72 g, 5.3 mmol) was added followed by copper iodide (CuI) (1.82 g, 9.6 mmol). The mixture was heated to 54 °C during 9 min. To the mixture was added propyne (5 wt% in THF, 2110 mL, 2.337 mol) during 19 min. This was followed by significant gas evolution. The mixture was stirred at 54–58 °C for 17 h. At this point HPLC indicated 98.9% conversion of starting material to product. To the mixture was added propyne (5 wt% in THF, 100 mL) and the mixture was stirred for 1 h at 54–56 °C. This resulted in no further conversion of starting material to product. To this mixture, ammonium chloride was added (NH_4_Cl) (25 wt% in water, 1200 mL). The mixture was transferred to a separatory funnel and the phases separated. The aqueous layer was extracted with 3 × 800 mL ethyl acetate (EtOAc). The pooled organic layers were dried over anhydrous sodium sulfate (Na_2_SO_4_) (242 g) and concentrated on a rotary evaporator (T,bath = 50 °C). The crude product was purified by flash column chromatography on silica (433 g). Eluent: heptanes/EtOAc 93:7 *v/v*). This gave compound **11** (11.21 g, 70 mmol, 69% yield, HPLC purity: 91.8%) as a red oil, which slowly crystallized.

*R*_f_ = 0.57 (EtOAc/heptane 2:8); m.p.: 36–38 °C; ^1^H NMR (400 MHz, CDCl_3_) δ 11.66 (s, 1H), 10.43 (s, 1H), 7.40 (t, *J* = 7.5 Hz, 1H), 7.00 (d, *J* = 7.5 Hz, 1H), 6.89 (d, *J* = 7.5 Hz, 1H), 2.12 (s, 3H); ^13^C{^1^H} NMR (101 MHz, CDCl_3_) δ 197.4, 162.4, 136.7, 128.6, 124.5, 112.0, 117.6, 93.9, 75.3, 4.7; IR (neat) cm^−1^: 3130 (br., O–H), 2877 (m, C(O)–H), 2757 (m, C(O)–H) 2229 (m, C≡C). 1651 (s, C=C); HRMS (ESI) *m/z* [M+H]^+^ calcd for C_10_H_9_O_2_: 161.0603, found: 161.0606.

### 3.3. 2-(Methoxymethoxy)-6-(prop-1-yn-1-yl)benzaldehyde *(**10**)*

In a 250 mL N_2_-flushed reactor equipped with mechanical stirrer, compound **11** (10.99 g, 68.6 mmol) was dissolved in dichloromethane (DCM) (water content: 50 ppm, 200 mL). To the mixture was added diisopropylethylamine (DIPEA) (water content: 720 ppm, 50 mL). The mixture was cooled to 3 °C. To the mixture was added MOM-Cl (11.0 mL, 143 mmol) for 3 min at a temperature of 3–5 °C. The mixture was heated to 41 °C and stirred at 40–41 °C for 3 h. At this point HPLC indicated 99.4% conversion of the starting material to product. The mixture was cooled to −3 °C and 1 M hydrochloric acid (50 mL) was added for 3 min. This caused the temperature to increase to 4 °C. The mixture was extracted with 3 × 300 mL EtOAc. The combined organic layers were washed with sodium hydrogen carbonate (NaHCO_3)_ (6 wt% in water, 550 mL) and then sodium chloride (25 wt% in water, 300 mL). The combined organic layers were dried over anhydrous magnesium sulfate (MgSO_4_) (48 g) and concentrated in vacuum (T,bath = 50 °C). The crude product was purified by flash column chromatography on silica (250 g). Eluent: heptanes/EtOAc 85:15 *v/v*). This gave **10** (11.69 g, 57.2 mmol, 84% yield, HPLC purity 99.0%) as an orange oil.

*R*_f_ = 0.35 (EtOAc/heptane 3:7); ^1^H NMR (400 MHz, CDCl_3_) δ 10.60 (s, 1H), 7.39 (t, *J* = 8.1 Hz, 1H), 7.15 (d, *J =* 7.1, 1H), 7.13 (d, *J =* 7.3, 1H), 5.27 (s, 2H), 3.50 (s, 3H), 2.12 (s, 3H); ^13^C{^1^H} NMR (101 MHz, CDCl_3_) δ 190.7, 158.5, 134.2, 127.53, 127.49, 126.3, 115.1, 95.0, 93.2, 77.1, 56.6, 4.9; IR (neat) cm^−1^: 2831 (m, C(O)–H), 2750 (m, C(O)–H), 2235 (m, C≡C), 1694 (s, C=O); HRMS (ESI) *m/z* [M+H]^+^ calcd for C_12_H_13_O_3_: 205.0865, found: 205.0874.

### 3.4. 8-(Methoxymethoxy)-3-methylisoquinoline *(**6**)*

In a 250 mL “mini-autoclave” (pressure tested to 10 bar), **10** (11.4 g, 55.8 mmol) and NH_3_ (7N in MeOH, 160 mL) were mixed. The reactor was sealed and submerged in an oil bath. The mixture was magnetically stirred at an external temperature of 65–76 °C for 16 h. At this point HPLC indicated 97.0% conversion of the starting material to product. The mixture was concentrated to dryness (final *p* = 41 mbar) in vacuum. The residue was mixed with potassium carbonate (K_2_CO_3_) (35 wt% in water, 180 mL) and EtOAc (350 mL). The layers were separated and the aqueous layer was extracted with 2 × 350 mL EtOAc. The combined organic layers were washed with sodium chloride (25 wt% in water, 480 mL) and dried over MgSO_4_ (28 g). The mixture was concentrated to dryness in vacuum (T,bath = 50 °C, final *p* = 36 mbar). The crude product was purified by flash column chromatography on silica (245 g). Eluent: heptanes/EtOAc 3:2 *v/v*). This gave **6** (10.04 g, 49.4 mmol, 89% yield, HPLC purity 98.8%) as yellowish crystals.

*R*_f_ = 0.33 (EtOAc/heptane 1:1); m.p.: 50–52 °C; ^1^H NMR (400 MHz, CDCl_3_) δ 9.57 (s, 1H), 7.56 (t, *J* = 8.0 Hz, 1H), 7.46 (s, 1H), 7.35 (d, *J* = 8.0 Hz, 1H), 7.11 (d, *J* = 8.0 Hz, 1H), 5.42 (s, 2H), 3.55 (s, 3H), 2.72 (s, 3H); ^13^C{^1^H} NMR (101 MHz, CDCl_3_) δ 154.3, 151.5, 146.8, 138.2, 131.7, 119.4, 119.2, 118.7, 108.4, 94.8, 56.6, 23.9; IR (neat) cm^−1^: 1629 (m, C=C); HRMS (ESI) *m/z* [M+H]^+^ calcd for C_12_H_14_NO_2_: 204.1025, found: 204.1028.

### 3.5. 3-Methylisoquinolin-8-ol *(**12**)*

In a 100 mL RB-flask with magnetic stirrer, oil bath and condenser, **6** (2.51 g, 12.3 mmol) and methanol (MeOH) (40 mL) were mixed. To the mixture was added hydrochloric acid (36 wt%, 2.75 mL). The mixture was heated at an external temperature of 69 °C for 2 h and then stirred at 15–25 °C for 16 h. At this point HPLC indicated 91.6% conversion of the starting material to product. The external temperature was raised to 70 °C and the mixture was stirred at this temperature for 3 h. At this point HPLC indicated 99.8% conversion of the starting material to product. The mixture was concentrated to dryness in a vacuum (T,bath = 50 °C, final *p* = 198 mbar). The residue was mixed with potassium hydrogen carbonate (KHCO_3_) (20 wt% in water, 50 mL) and extracted with EtOAc (3 × 150 mL). The combined organic layers were washed with sodium chloride (25 wt% in water, 50 mL) and dried over Na_2_SO_4_ (42 g). The mixture was concentrated to dryness in a vacuum (final *p* = 29 mbar). The crude product was stored in the freezer.

In a 250 mL 3-necked reactor with mechanical stirrer, heating mantle and condenser, **7** (11.0 g, 54.1 mmol) and MeOH (180 mL) were mixed. To the mixture was added hydrochloric acid (36 wt%, 12.0 mL). The mixture was heated at ~50 °C for 16 h. At this point HPLC indicated 99.9% conversion of the starting material to product. The mixture was concentrated to dryness in a vacuum (T,bath = 50 °C, final *p* = 205 mbar). The residue was mixed with KHCO_3_ (20 wt% in water, 220 mL) and extracted with EtOAc (3 × 650 mL). The combined organic layers were washed with sodium chloride (25 wt% in water, 350 mL) and dried over Na_2_SO_4_ (103 g). The mixture was concentrated to dryness in a vacuum (final *p* = 26 mbar). The crude product was combined with the crude product from above and purified by flash column chromatography on silica (271 g). Eluent: DCM/MeOH 9:1 *v/v*. This gave **12** (9.52 g, 59.8 mmol, 90% yield combined, HPLC purity 99.7%) as brown crystals.

*R*_f_ = 0.54 (CH_2_Cl_2_/MeOH 9:1); m.p.: 202–203 °C; ^1^H NMR (400 MHz, DMSO-*d*_6_) δ 10.59 (s, 1H), 9.35 (s, 1H), 7.52–7.46 (m, 2H), 7.23 (d, *J* = 8.2 Hz, 1H), 6.88 (d, *J* = 7.6 Hz, 1H), 2.57 (s, 3H); ^13^C{^1^H} NMR (101 MHz, DMSO-*d*_6_) δ 154.5, 151.4, 146.8, 137.4, 131.5, 118.1, 117.6, 116.1, 108.8, 23.8; IR (neat) cm^−1^: 1633 (m, C=C); HRMS (ESI) *m/z* [M+H]^+^ calcd for C_10_H_10_NO: 160.0762, found: 160.0753.

### 3.6. Ethyl 2-((3-methylisoquinolin-8-yl)oxy)acetate *(**9**)*

In a 250 mL 3-necked reactor equipped with mechanical stirrer and flushed with N_2_, **12** (7.5 g, 47.1 mmol) and dimethylformamide (DMF) (96 mL) were mixed. The mixture was cooled to 0 °C and sodium ethanoate (NaOEt) (3.54 g, 52.0 mmol) was added. The mixture was stirred at ~0 °C for 1 h. To the mixture was added ethyl bromoacetate (5.7 mL, 51.4 mmol) during 6 min at ~0 °C. The mixture was allowed to heat to 20–22 °C and stirred at this temperature for 19 h at which point no further conversion of the starting material was detected by HPLC. The mixture was concentrated to dryness in vacuum (T,bath = 50 °C, final *p* = 0 mbar). The residue was mixed with 340 mL water and 680 mL EtOAc and the phases separated. The aqueous layer was extracted with EtOAc (2 × 680 mL). The combined organic layers were washed with water (190 mL) and concentrated to dryness (final *p* = 0 mbar). The crude product was purified by flash column chromatography on silica (152 g). Eluent: DCM/MeOH 100:5 *v/v*). This gave **6** (6.27 g, HPLC purity 96.3%) as brownish crystals and 2.36 g **12**, which was again subjected to the reaction conditions and purification. This provided additional **6** (2.13 g, HPLC purity 97.1%) resulting in a total of 8.40 g **9** (34.2 mmol, 73% yield)

An analytical sample was dissolved in ethanol (EtOH) (1 mL), 3N NaOH (0.3 mL, 0.9 mmol) was added and the mixture was stirred at 22 °C for 1 h. The mixture was concentrated in vacuo, redissolved in water/acetone (1:2, 1.5 mL), and acidified to pH 3 with 37% aqueous HCl. The precipitate was collected by filtration and washed with water and acetone to give 2-((3-methylisoquinolin-8-yl)oxy)acetic acid as a pale yellow solid (0.12 g), which was characterized. *R*_f_ = 0.64 (acetone/AcOH/water 6:3:1); m.p.: 211–213 °C; ^1^H NMR (400 MHz, D_2_O) δ 9.70 (s, 1H), 8.09 (s, 1H), 8.04 (t, *J* = 8.0 Hz, 1H), 7.67 (d, *J* = 8.0 Hz, 1H), 7.21 (d, *J* = 8.0 Hz, 1H), 5.01 (s, 2H), 2.81 (s, 3H); ^13^C{^1^H} NMR (101 MHz, D_2_O) δ 172.7, 155.7, 142.8, 141.7, 140.3, 138.5, 123.7, 119.2, 118.3, 109.1, 66.0, 18.2; IR (neat) cm^−1^: 1722 (m, C=O), 1654 (s, C=C); HRMS (ESI) *m/z* [M+H]^+^ calcd for C_12_H_12_NO_3_: 218.0817, found: 218.0815.

### 3.7. 7-Methylfuro[3,2-h]isoquinolin-3(2H)-one *(**8**)*

In a 100 mL RB-flask with magnetic stirrer, **9** (3.1 g, 12.7 mmol) was dissolved in EtOH (32 mL). To the solution was added NaOH (3 N in water, 7.0 mL). The mixture was stirred at ~22 °C for 1 h at which point HPLC indicated >99.9% conversion of the starting material. The mixture was concentrated to dryness in vacuum (T,bath = 40 °C, final *p* = 94 mbar). The residue was redissolved in acetone/water (2:1 *v/v*, 38 mL). To the mixture was added hydrochloric acid (36 wt%, 2.0 mL). At this point the pH of the mixture was ~3. The mixture was concentrated to dryness in vacuum (T,bath = 40 °C, final *p* = 32 mbar). The residue was evaporated with DCM (2 × 40 mL) to remove residual water and kept at high vacuum (oil pump) for 86 min. The residue was mixed with SOCl_2_ (170 mL) in a 250 mL 3-necked reactor. The temperature was increased to 70 °C and the mixture was magnetically stirred at this temperature for 1 h. The mixture was concentrated to dryness (T,bath = 50 °C, final *p* = 105 mbar). The residue was mixed with 90 mL DCM and stirred magnetically for 15 min at ~22 °C. Salts were apparent in the mixture. To the mixture was added AlCl_3_ (16.8 g, 126 mmol) and the mixture was stirred at rt for 30 min. At this point HPLC indicated 99.0% conversion of the starting material into product. The mixture was added to NaHCO_3_ (6 wt% in water, 1.0 L) at 12–17 °C during 10 min. To the mixture was added methyl tert-butyl ether (MTBE) (300 mL) and the resulting emulsion was filtered on a G-3 glass filter. The two-phase filtrate was transferred to a separatory funnel and the layers separated. The aqueous layer was extracted with 2 × 300 mL MTBE. The pooled organic extracts were filtered and concentrated to a volume of ~200 mL in vacuum (T,bath = 40 °C) and then passed through silica (20 g) with MTBE (800 mL). The filtrate was concentrated to dryness in vacuum (T,bath = 40 °C, final *p* = 32 mbar). This gave **8** (1.98 g, 9.9 mmol, 79% yield, HPLC purity 96.7%) as a brown solid.

*R*_f_ = 0.7 (CH_2_Cl_2_/ MeOH 95:5); m.p.: 120–122 °C (Lit. [13] 119–122 °C); ^1^H NMR (400 MHz, CDCl_3_-*d*) δ 9.54 (s, 1H), 7.75 (d, *J* = 8.5 Hz, 1H), 7.56 (s, 1H), 7.35 (d, *J* = 8.5 Hz, 1H), 4.88 (s, 2H), 2.77 (s, 3H); ^13^C{^1^H} NMR (101 MHz, CDCl_3_) δ 197.6, 174.9, 157.2, 146.3, 142.4, 124.3, 120.7, 119.9, 117.2, 115.5, 76.3, 24.6; IR (neat) cm^−1^: 1697 (s, C=O), 1634 (s, C=C); HRMS (ESI) *m/z* [M+H]^+^ calcd for C_12_H_10_NO_2_: 200.0712, found: 200.0716. ^1^H NMR spectroscopic data was consistent with the literature [13].

### 3.8. TMC-120B *(**1**)*

In a 1 L 3-necked reactor with mechanical stirrer, condenser, heating mantle and addition funnel, acetone (210 mL) and p-toluene-sulfonic acid (*p*-TsOH) (4.58 g, 24.1 mmol) were mixed. The reactor was evacuated (*p* < 300 mbar) and backfilled with N_2_ three times. The temperature was increased to 56 °C (reflux). To the mixture was added a mixture of **8** (3.5 g, 17.6 mmol), dichloroethane (DCE) (210 mL) and acetone (40 mL) (degassed with N_2_) during 2 h and 10 min while the temperature was gradually increased to keep the mixture at reflux. The mixture was stirred for 2 h at ~67 °C and then NaHCO_3_ (6 wt% in water, 500 mL) was added. The mixture was extracted with EtOAc (3 × 800 mL), washed with sodium chloride (25 wt% in water, 200 mL) and dried over MgSO_4_ (54 g). The mixture was concentrated to dryness in vacuum (T,bath = 40 °C, final *p* = 44 mbar). The crude product was purified by flash column chromatography on silica (520 g. Eluent: DCM/EtOAc 1:0 -> 100:5 -> 9:1 *v/v*). This gave **1** as a yellow solid (1.11 g, HPLC purity: 99.1%) and **1** as a yellow solid (0.35 g, HPLC purity: 98.3%) from less pure fractions, resulting in a total of 1.46 g **1** (6.1 mmol, 35% yield).

*R*_f_ = 0.50 (CH_2_Cl_2_/MeOH 98:2); m.p.: 174–176 °C (Lit. [13] 175–178 °C); ^1^H NMR (400 MHz, CDCl_3_) δ 9.61 (s, 1H), 7.92 (d, *J* = 8.5 Hz, 1H), 7.65 (s, 1H), 7.44 (d, *J* = 8.5 Hz, 1H), 2.84 (s, 3H), 2.45 (s, 3H), 2.26 (s, 3H); ^13^C{^1^H} NMR (101 MHz, CDCl_3_) δ 181.9, 164.0, 154.4, 145.7, 144.9, 141.9, 135.2, 126.1, 121.1, 120.7, 120.2, 114.8, 23.5, 20.6, 17.8. IR (neat) cm^−1^: 1694 (s, C=O), 1657 (s, C=C); HRMS (ESI) *m/z* [M+H]^+^ calcd for C_15_H_14_NO_2_: 240.1025, found: 240.1013. ^1^H and ^13^C NMR spectroscopic data was consistent with those reported in the literature [1,13].

## 4. Conclusions

A concise total synthesis of the fungal natural product TMC-120B (**1**) has been presented. With an overall yield of 9% over seven steps to access **1**, this presents a significant improvement over previously reported total syntheses with 2.9% overall yield over 11 steps. The developed synthetic route enables more efficient access to the TMC-120B alkaloid for further biological evaluation in pursue of improved treatments for epilepsy. Such studies are currently undergoing and the results will be disclosed in due course.

## Data Availability

Data is contained within the article.

## Supplementary Materials

Copies of NMR and IR spectra for compounds **1**, **5**, hydrolyzed **6**, and **7**–**10**.
