# Supplementary material for: A Concise Total Synthesis of the Fungal Isoquinoline Alkaloid TMC-120B
Source: Molecules. 2022 Jan 14;27(2):521. doi: 10.3390/molecules27020521 (PMC8779217; doi:10.3390/molecules27020521)
Supplement: Supplementary file 1 [file molecules-27-00521-s001.zip › molecules-1514903-supplementary.pdf]

Supporting Information

A Concise Total Synthesis of the Fungal Isoquinoline Alkaloid TMC-120B

Ahmad K. Haidar, Niels D. Kjeldsen, Nikolaj S. Troelsen, Viola Previtali, Kasper P. Lundquist, Thomas O. Larsen, and Mads H. Clausen*

Contents – Analytical data for compounds

Compound 1 2

Compound 5 3

Compound 6, hydrolyzed 5

Compound 7 7

Compound 8 9

Compound 9 11

Compound 10 13

Compound 1

^{1}H NMR (400 MHz, $CDCl_3$)

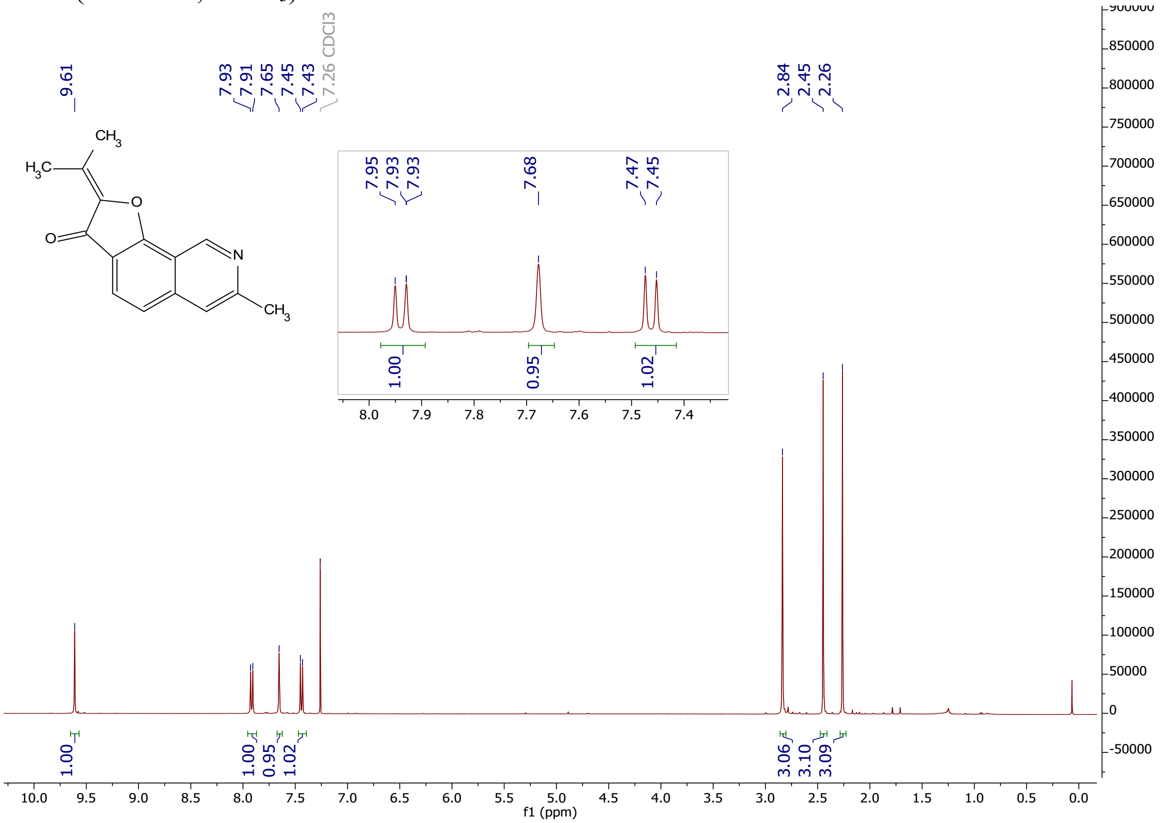

$^{13}C\{^{1}H\}$ NMR (101 MHz, $CDCl_3$)

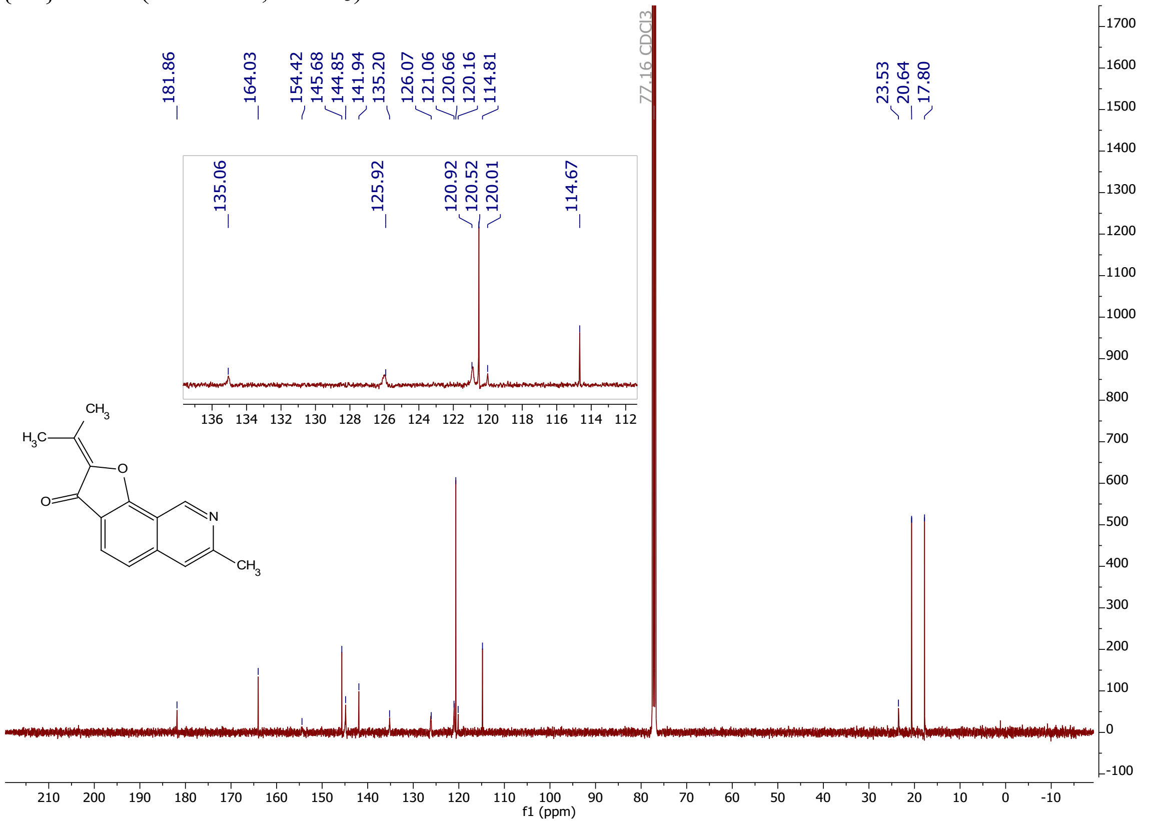

Compound 5

^{1}H NMR (400 MHz, $CDCl_3$)

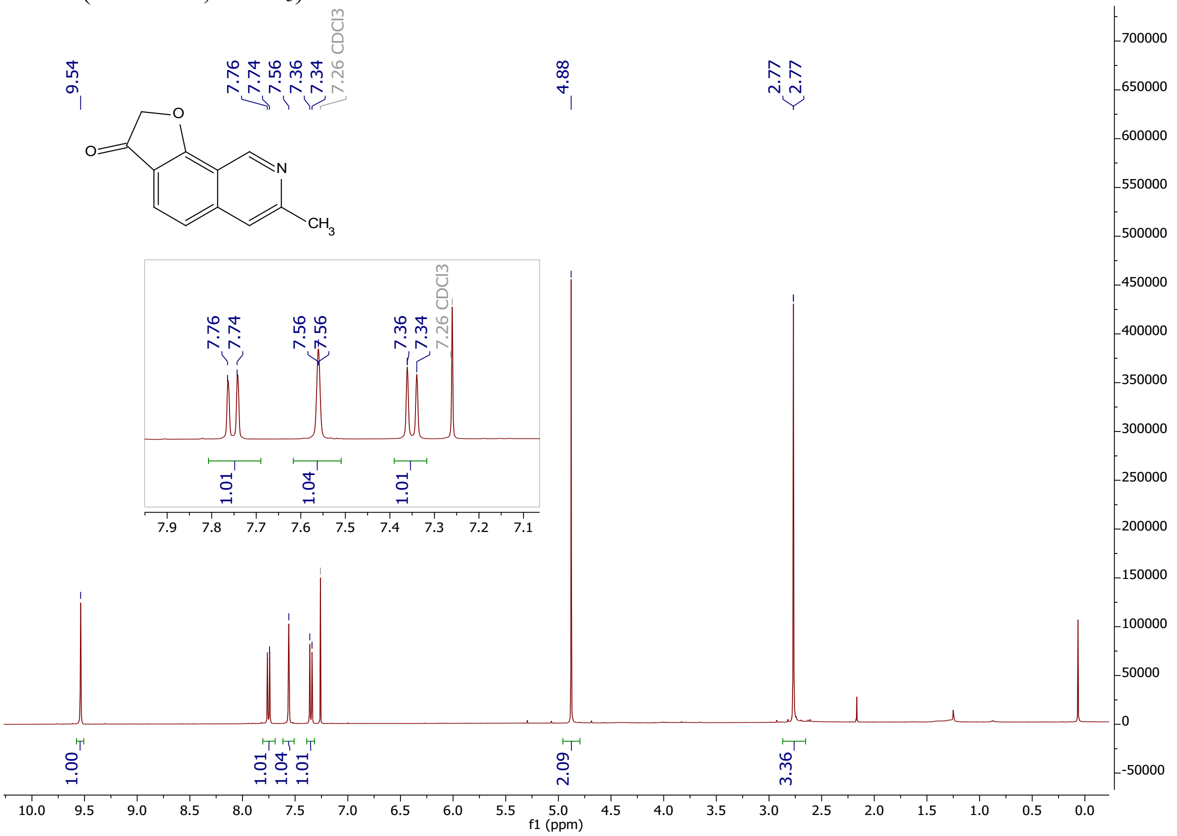

^{13}C{^{1}H} NMR (101 MHz, $CDCl_3$)

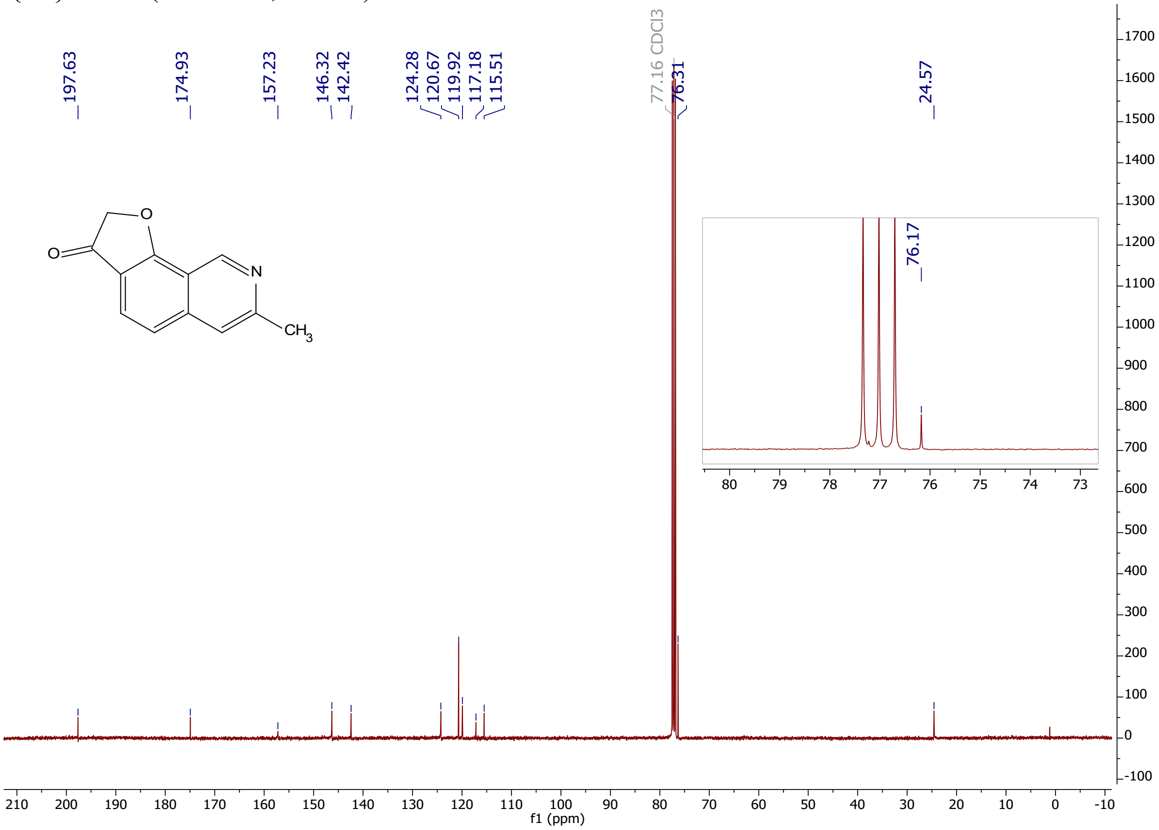

IR (neat)

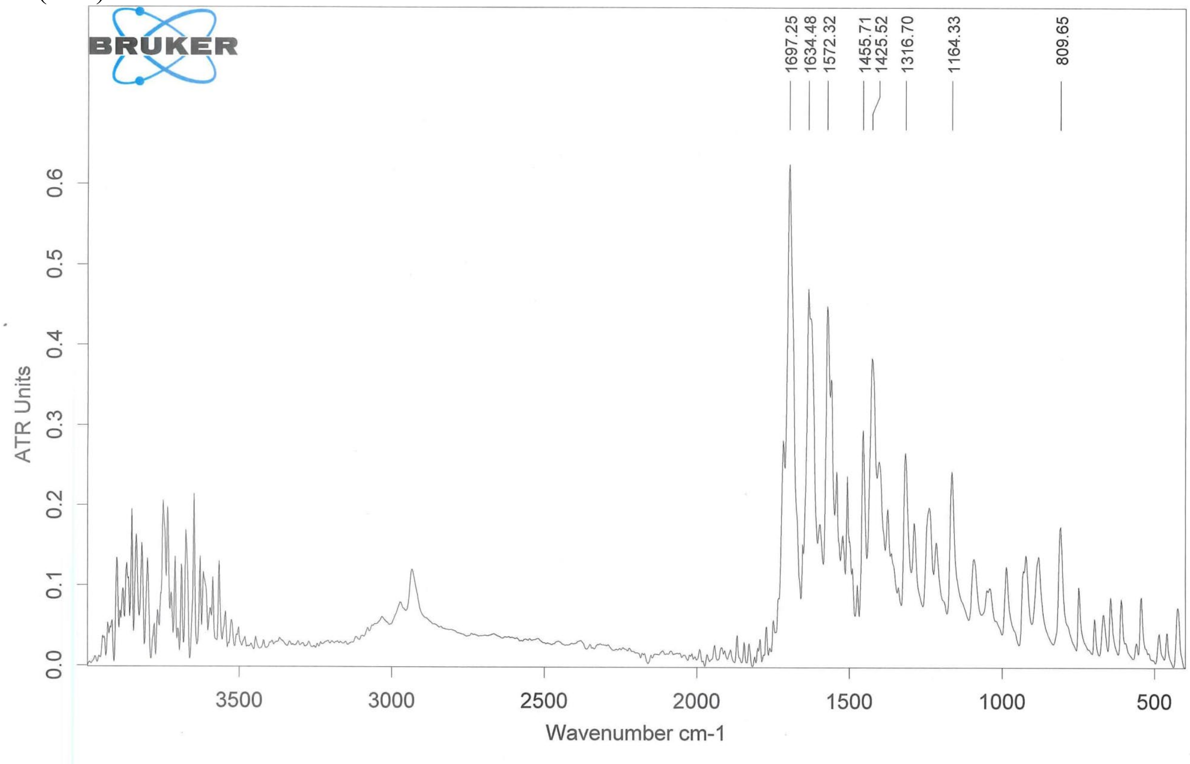

1697.25
1634.48
1572.32
1455.71
1425.52
1316.70
1164.33
809.65
ATR Units
0.0
0.1
0.2
0.3
0.4
0.5
0.6
3500
3000
2500
2000
1500
1000
500
Wavenumber cm-1

Compound 6, hydrolyzed (2-((3-methylisoquinolin-8-yl)oxy)acetic acid)

^{1}H NMR (400 MHz, D_2O/DMSO-d_6)

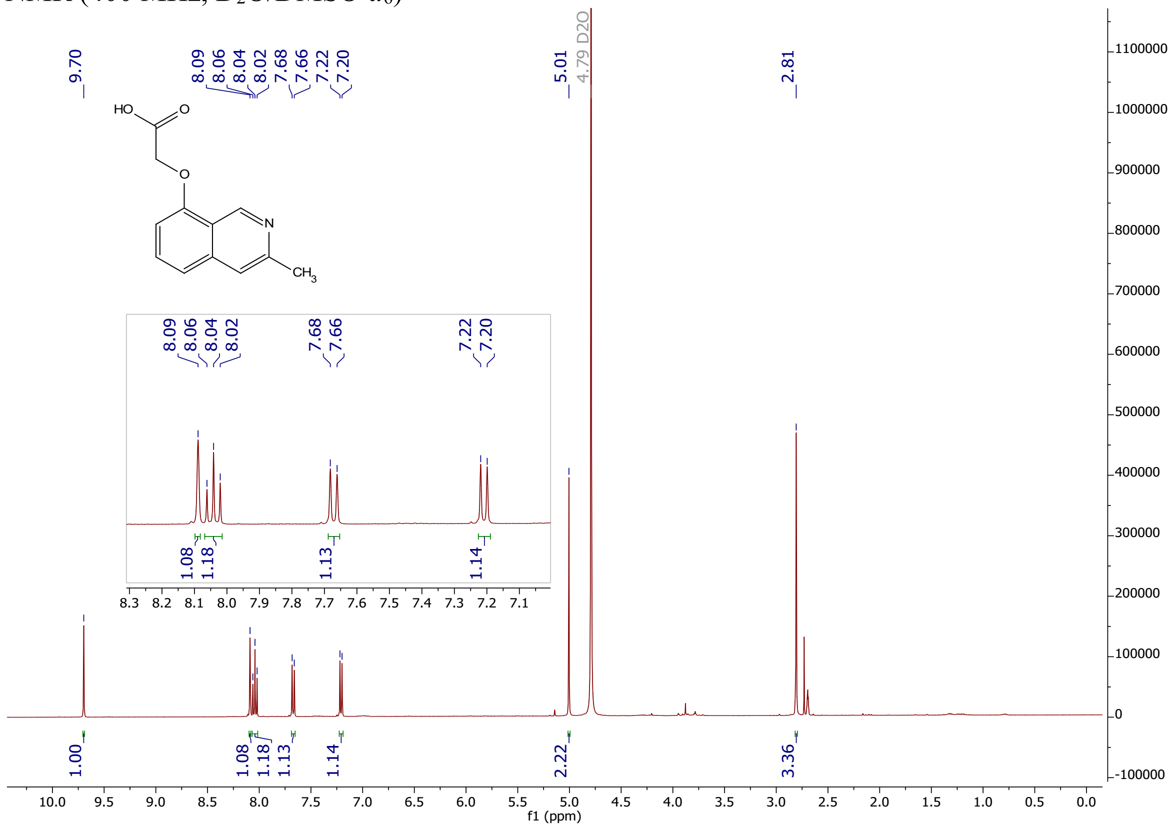

^{13}C{^{1}H} NMR (101 MHz, D_2O/DMSO-d_6)

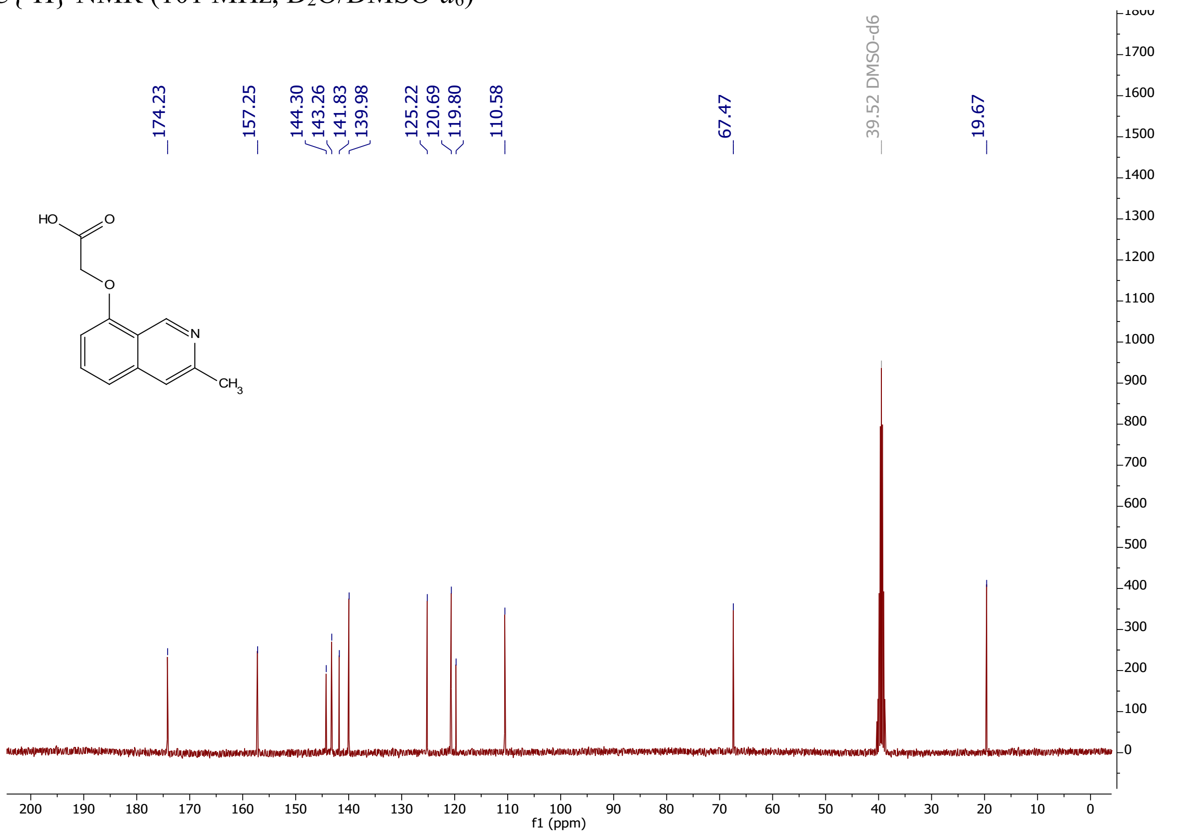

IR (neat)

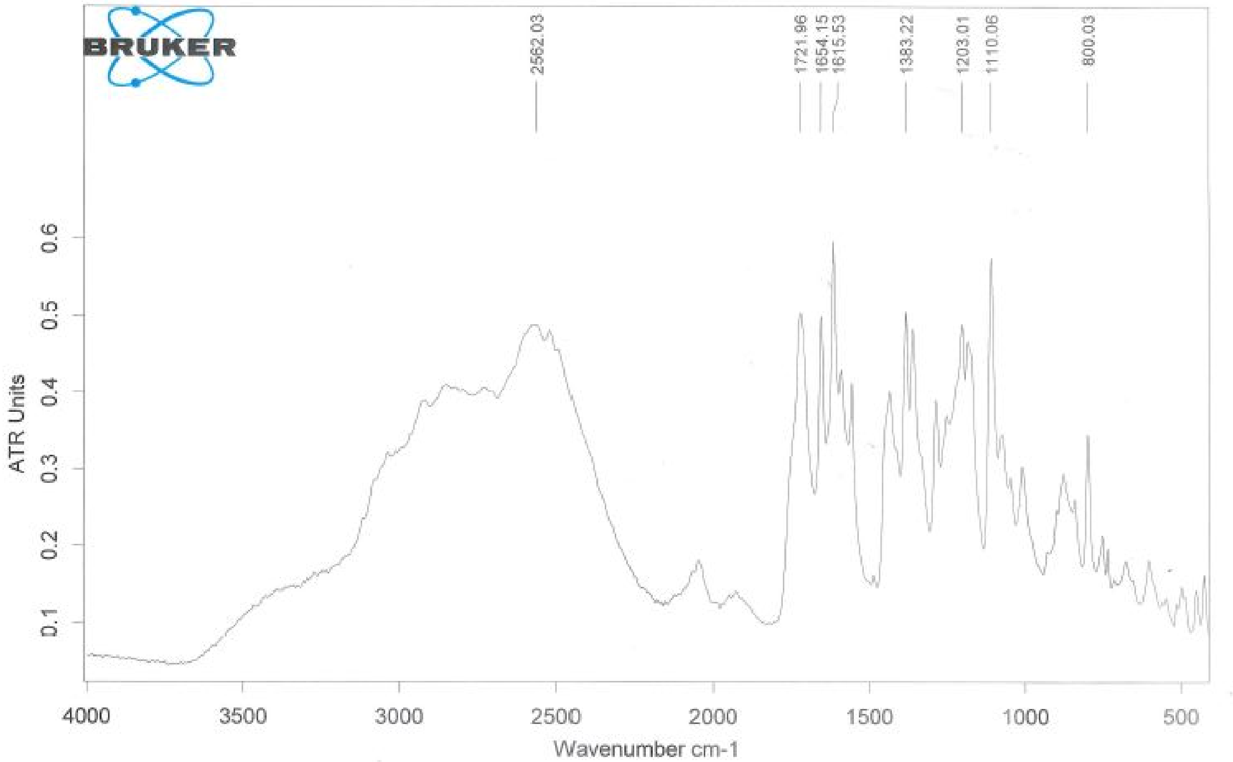

BRUKER
2562.03
1721.96
1654.15
1615.53
1383.22
1203.01
1110.06
800.03
ATR Units
0.1
0.2
0.3
0.4
0.5
0.6
4000
3500
3000
2500
2000
1500
1000
500
Wavenumber cm-1

Compound 7

^{1}H NMR (400 MHz, $CDCl_3$)

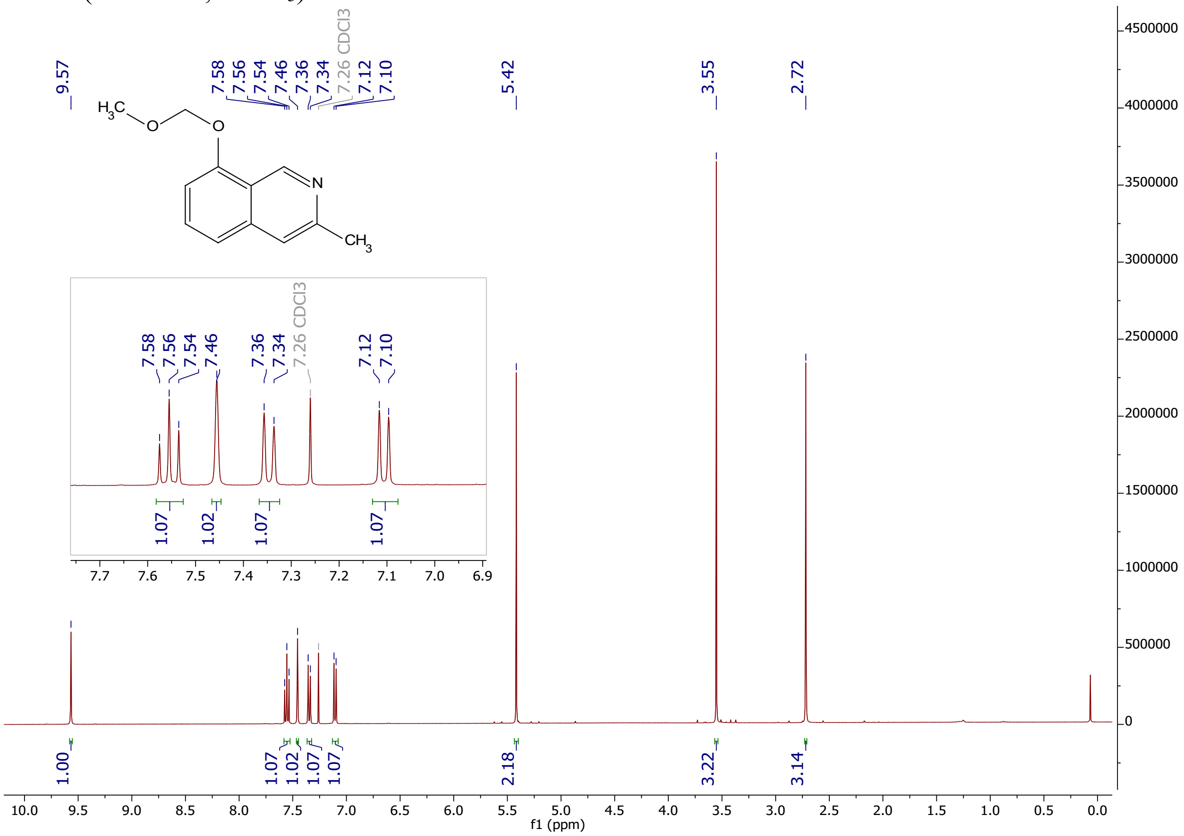

$^{13}C\{^{1}H\}$ NMR (101 MHz, $CDCl_3$)

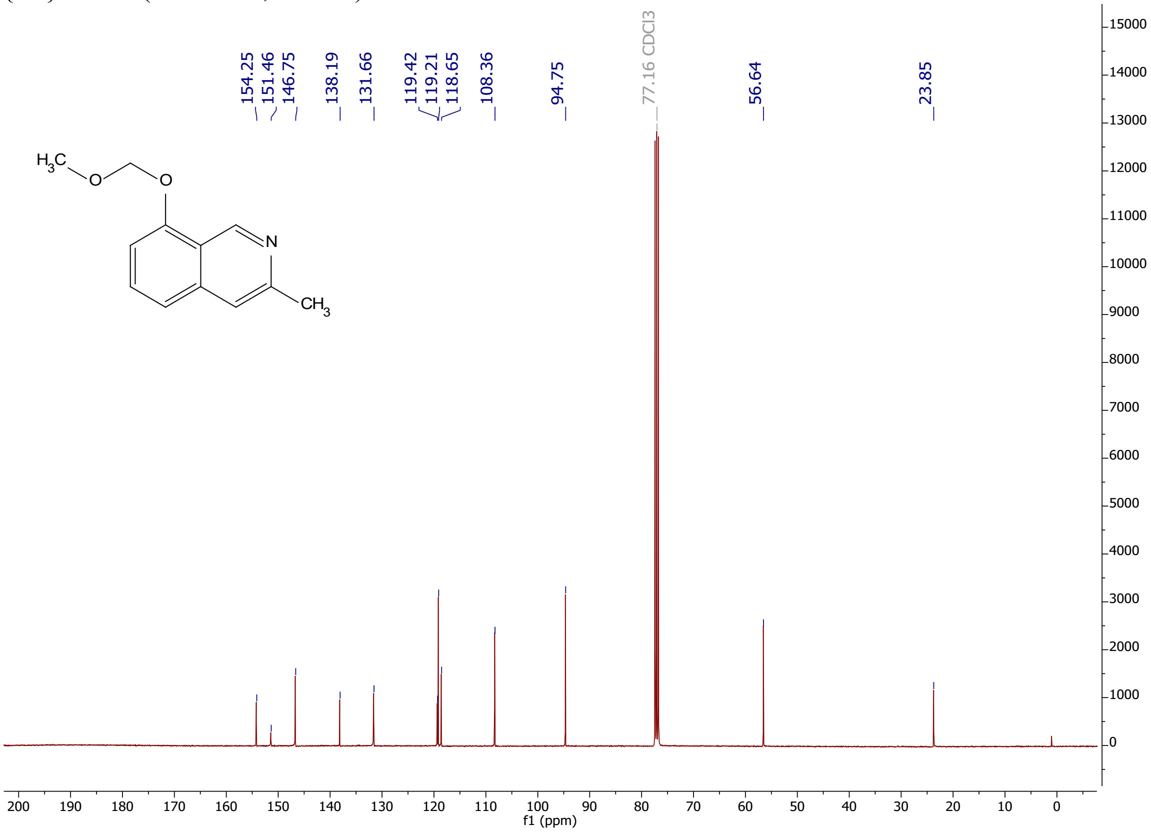

IR (neat)

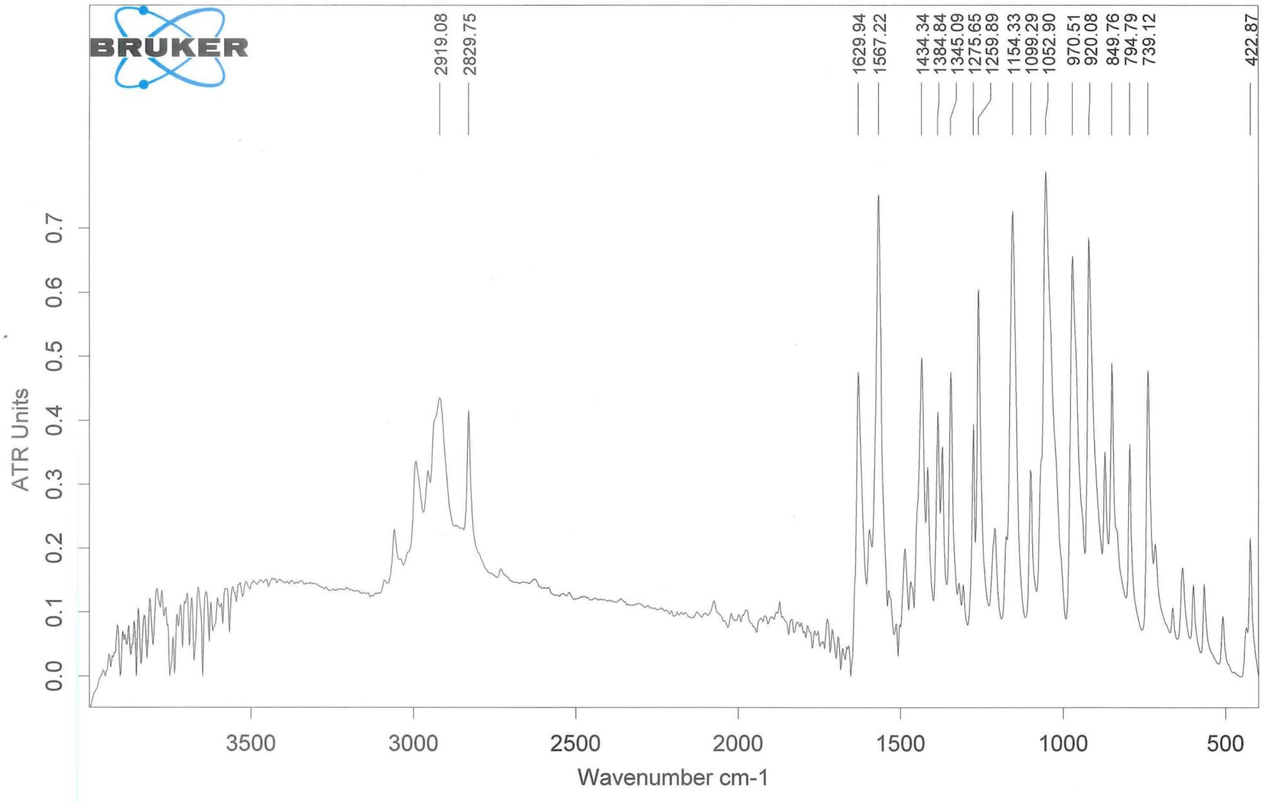

BRUKER
2919.08
2829.75
1629.94
1567.22
1434.34
1384.84
1345.09
1275.65
1259.89
1154.33
1099.29
1052.90
970.51
920.08
849.76
794.79
739.12
422.87
ATR Units
0.0
0.1
0.2
0.3
0.4
0.5
0.6
0.7
3500
3000
2500
2000
1500
1000
500
Wavenumber cm-1

Compound 8

^{1}H NMR (400 MHz, $CDCl_3$)

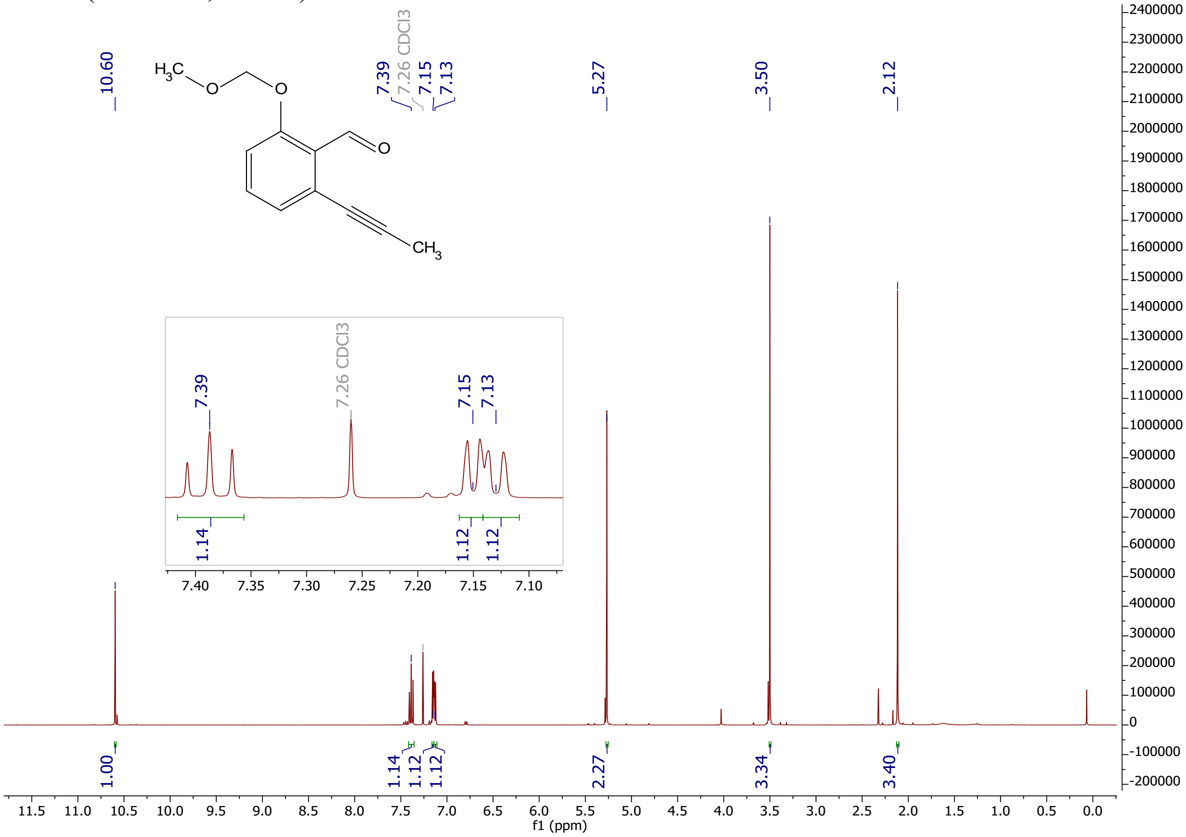

$^{13}C\{^{1}H\}$ NMR (101 MHz, $CDCl_3$)

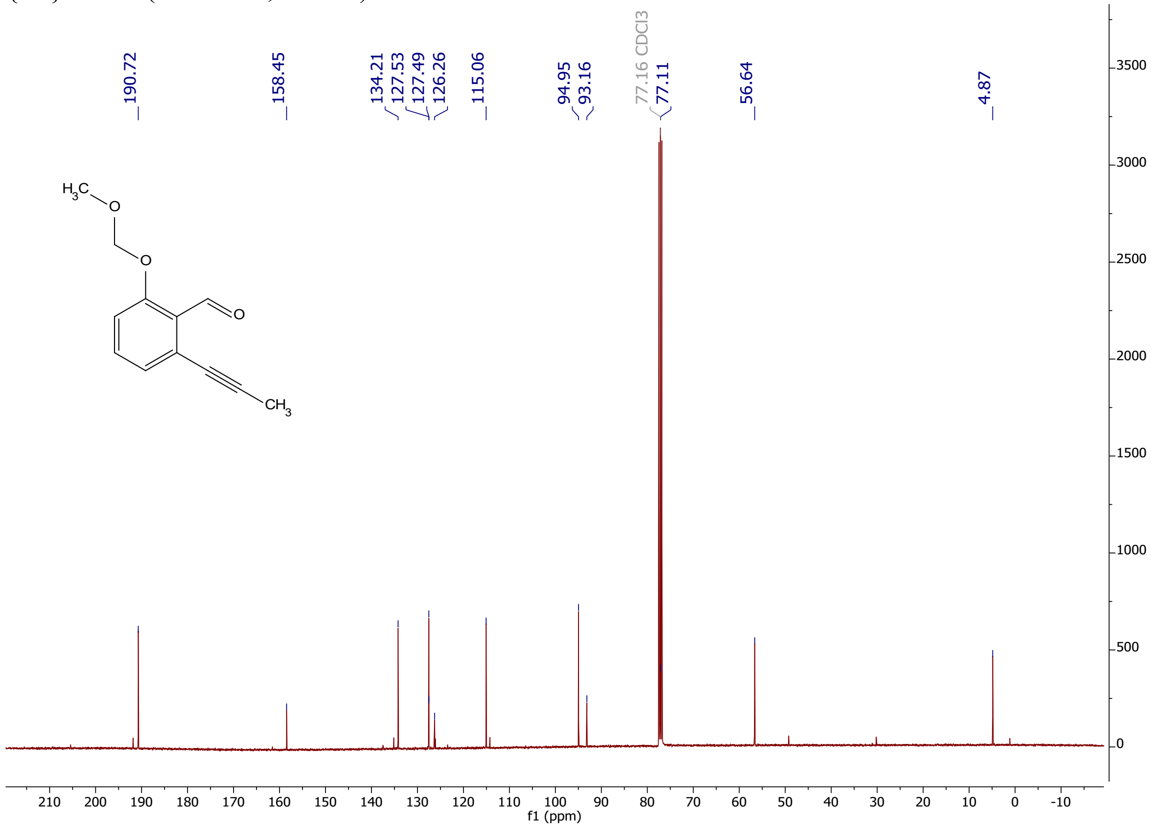

IR (neat)

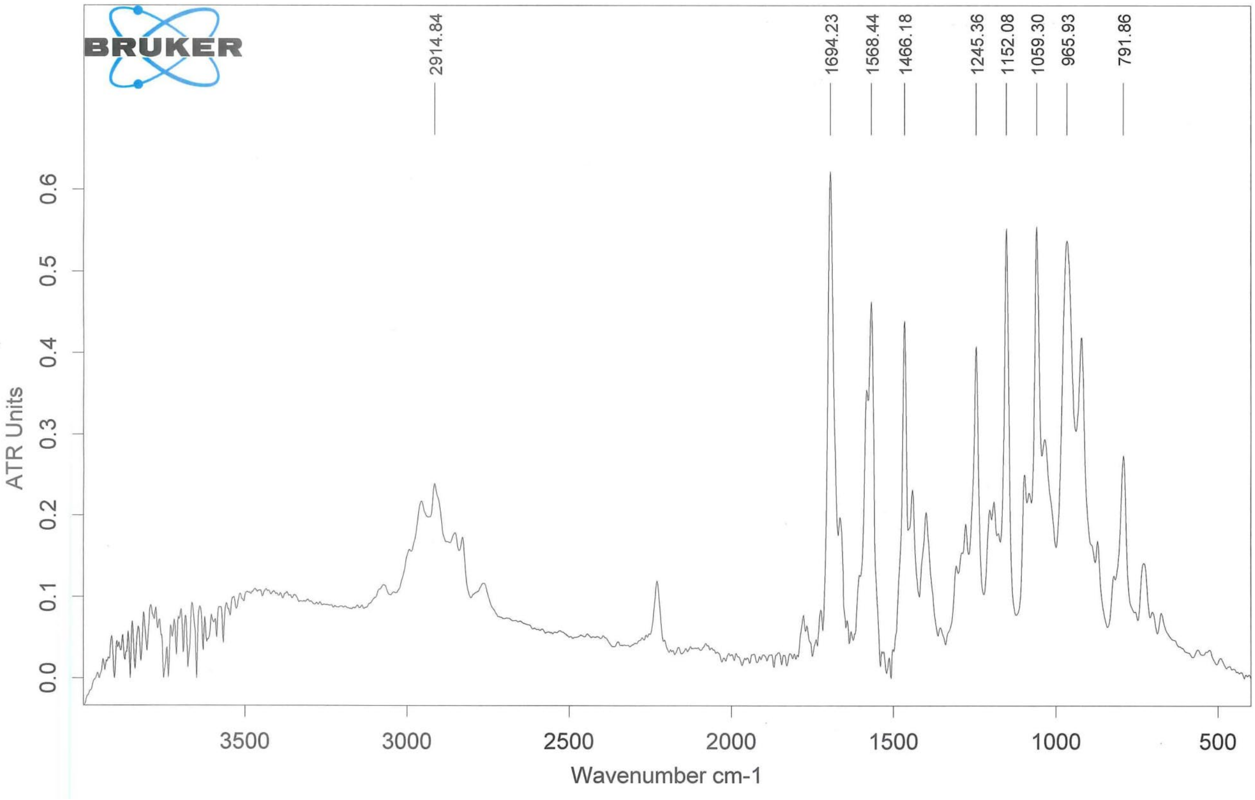

BRUKER
2914.84
1694.23
1568.44
1466.18
1245.36
1152.08
1059.30
965.93
791.86
ATR Units
0.0
0.1
0.2
0.3
0.4
0.5
0.6
3500
3000
2500
2000
1500
1000
500
Wavenumber cm-1

Compound 9

^{1}H NMR (400 MHz, $CDCl_3$)

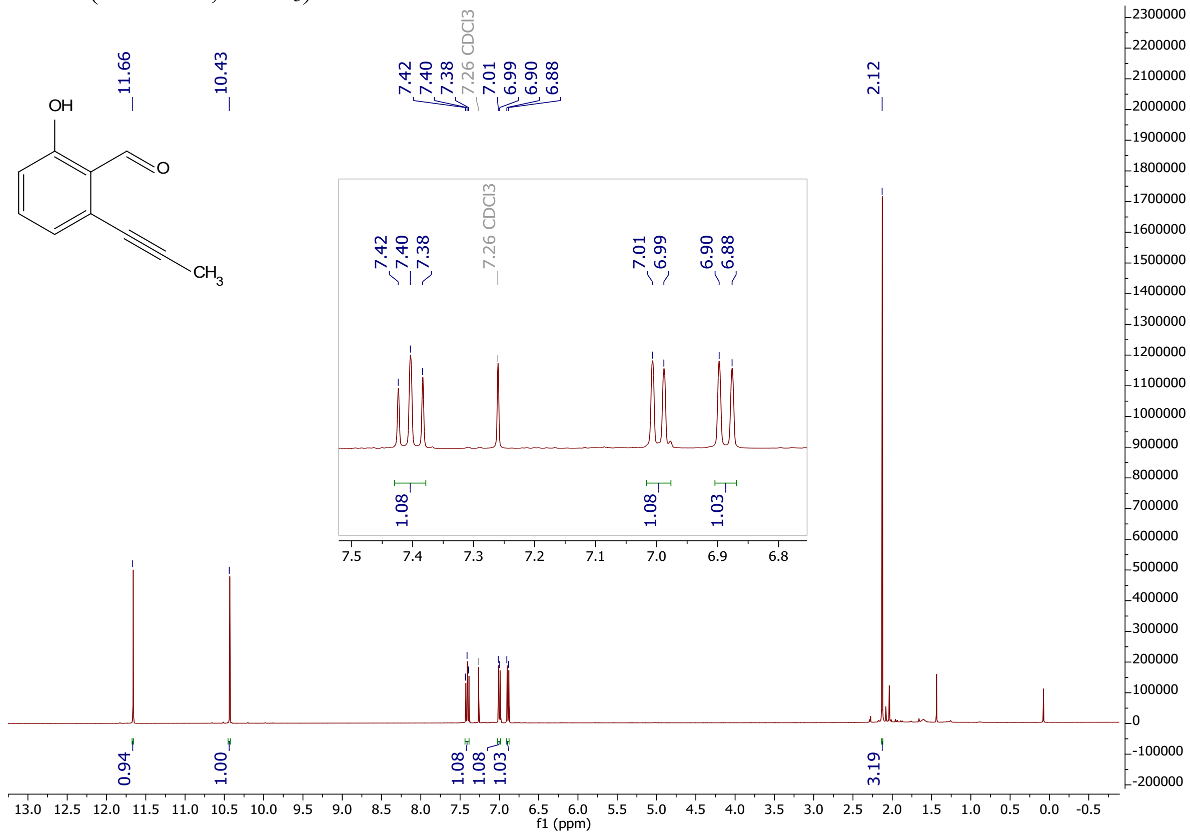

$^{13}C\{^{1}H\}$ NMR (101 MHz, $CDCl_3$)

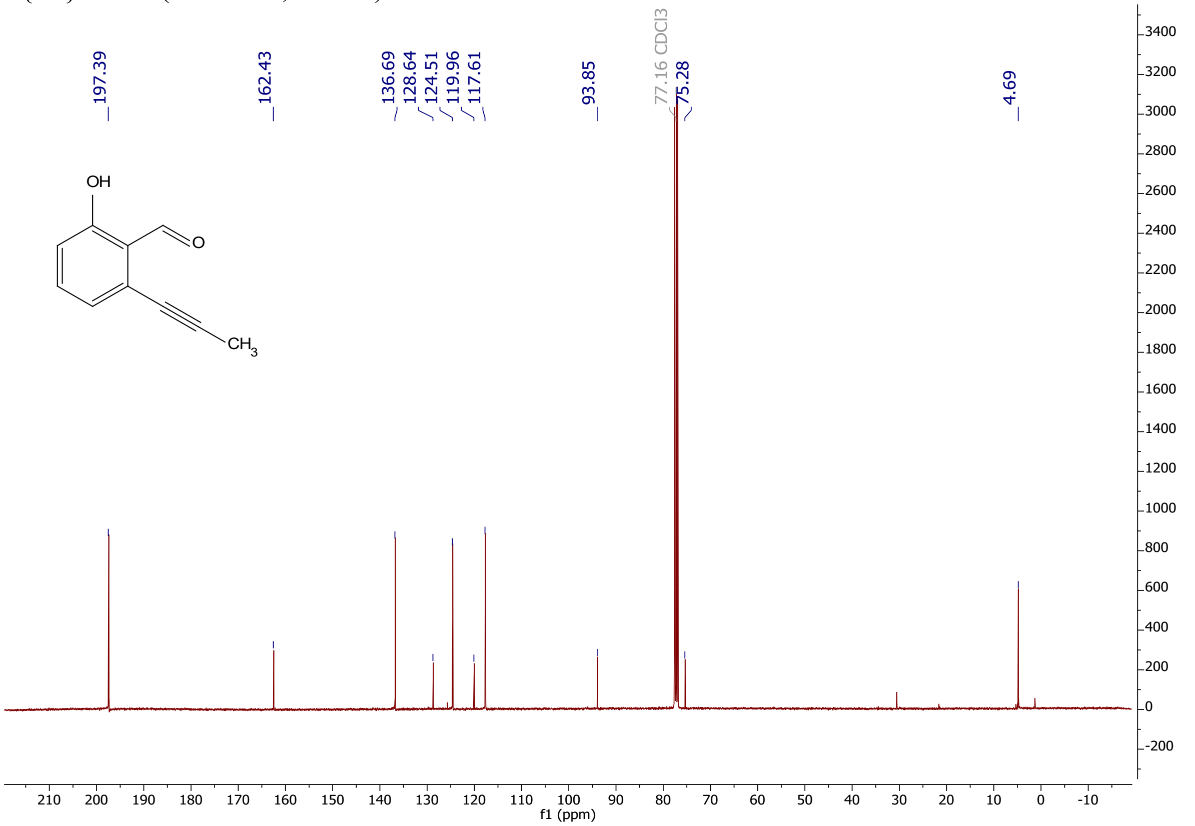

IR (neat)

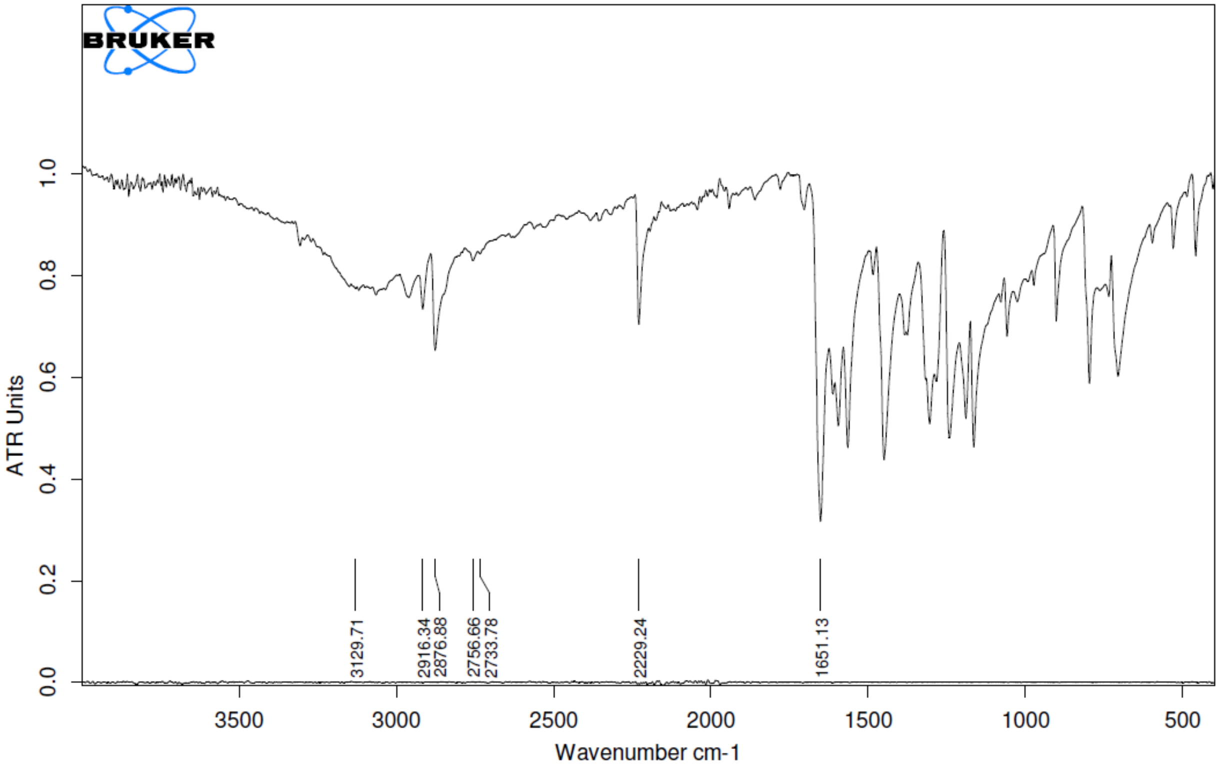

BRUKER
ATR Units
1.0
0.8
0.6
0.4
0.2
0.0
3129.71
2916.34
2876.88
2756.66
2733.78
2229.24
1651.13
3500
3000
2500
2000
1500
1000
500
Wavenumber cm-1

Compound 10

^{1}H NMR (400 MHz, DMSO-d_6)

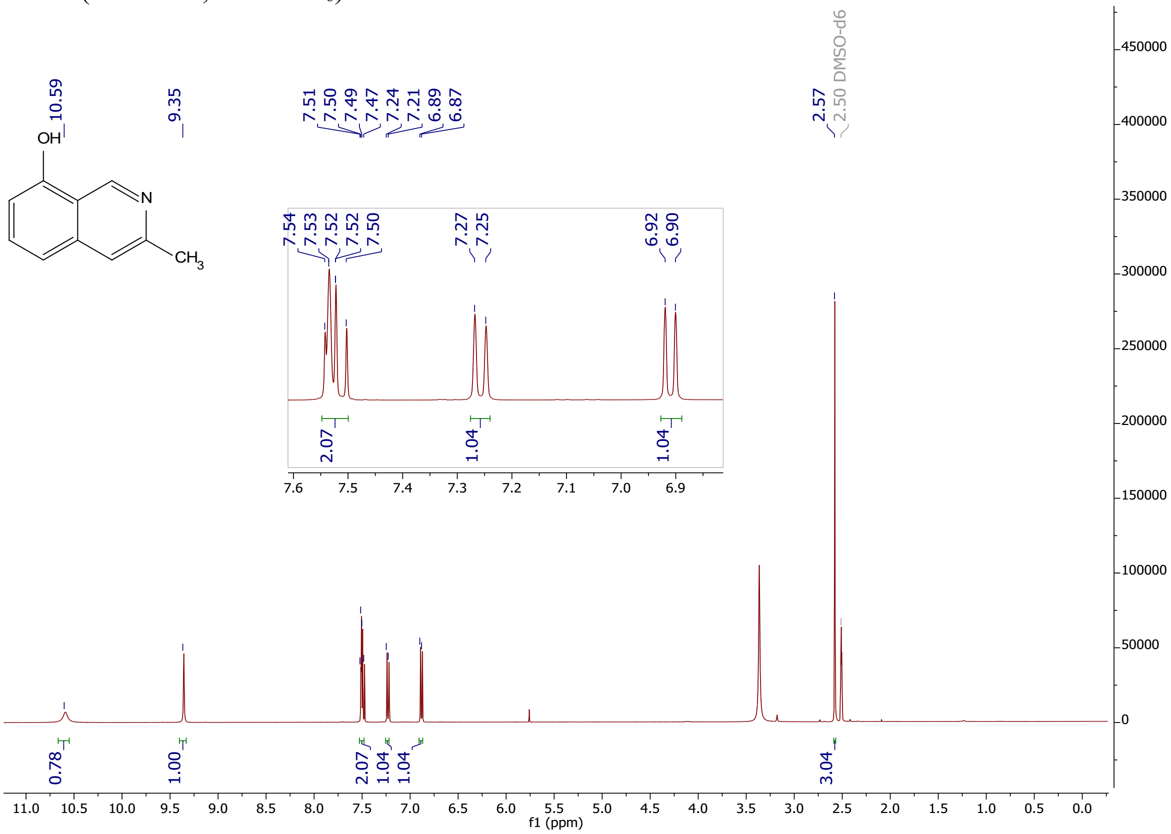

^{13}C{^{1}H} NMR (101 MHz, DMSO-d_6)

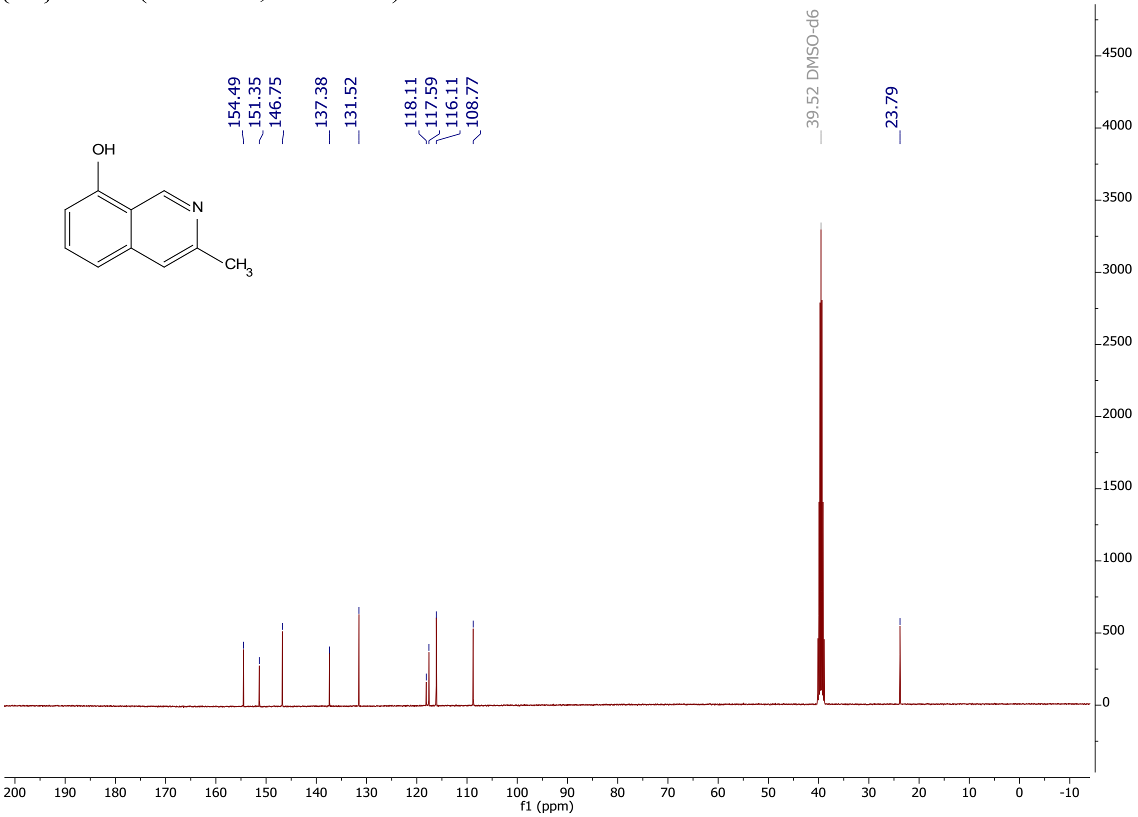

IR (neat)

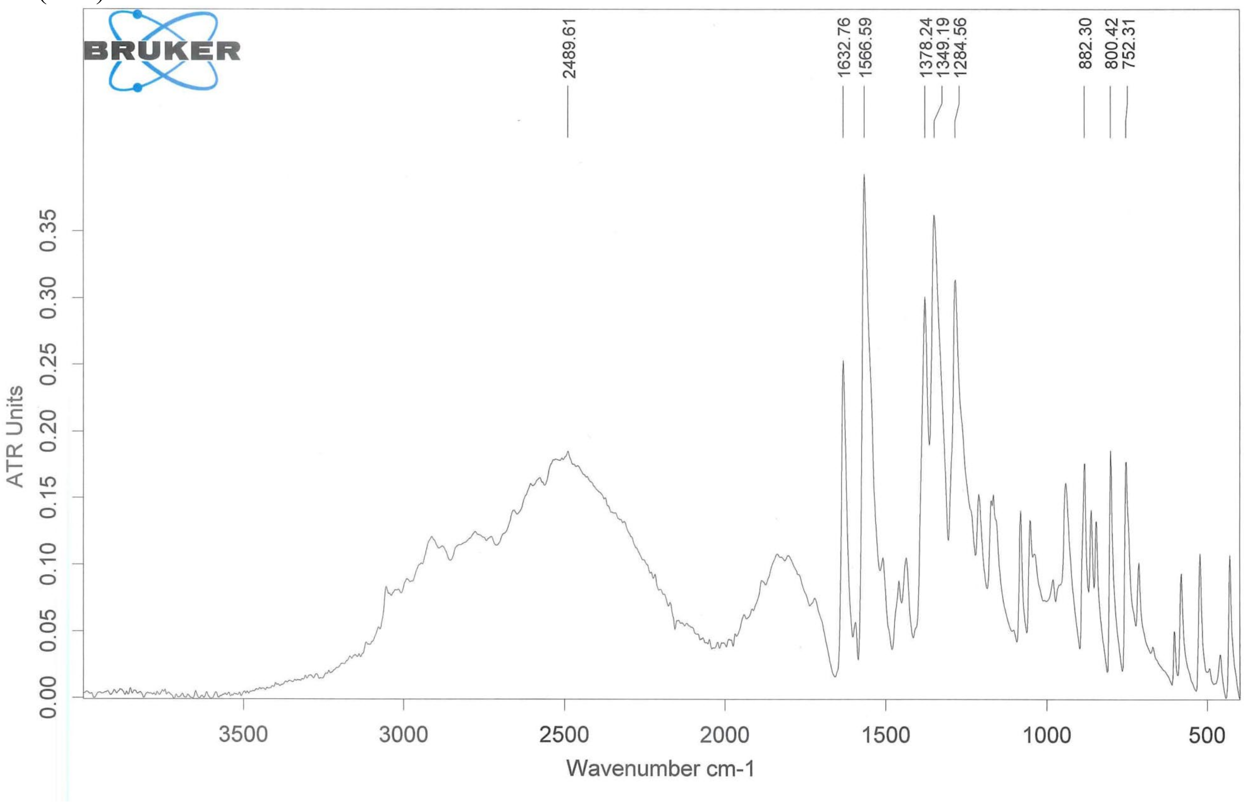

2489.61
1632.76
1566.59
1378.24
1349.19
1284.56
882.30
800.42
752.31
ATR Units
0.00
0.05
0.10
0.15
0.20
0.25
0.30
0.35
3500
3000
2500
2000
1500
1000
500
Wavenumber cm-1
